# Detecting cell types and densities in the tumor microenvironment improves prognostic risk assessment for breast cancer

**DOI:** 10.17305/bb.2024.10974

**Published:** 2024-08-16

**Authors:** Pu Liu, Xueli Zhang, Wenwen Wang, Yunping Zhu, Yongfang Xie, Yanhong Tai, Jie Ma

**Affiliations:** 1 Chongqing Key Laboratory on Big Data for Bio Intelligence, Chongqing University of Posts and Telecommunications, Chongqing, China; 2 State Key Laboratory of Medical Proteomics, Beijing Proteome Research Center, National Center for Protein Sciences (Beijing), Beijing Institute of Lifeomics, Beijing, China; 3 Department of Pathology, The Fifth Medical Centre of Chinese PLA General Hospital, Beijing, China; 4 Department of Dermatology, Peking Union Medical College Hospital, Chinese Academy of Medical Sciences and Peking Union Medical College, Beijing, China

**Keywords:** Tumor microenvironment (TME), transfer learning, breast cancer, artificial intelligence, deep convolutional neural network (CNN)

## Abstract

A comprehensive evaluation of the relationship between the densities of various cell types in the breast cancer tumor microenvironment (TME) and patient prognosis is currently lacking. Additionally, the absence of a large patch-level whole slide imaging (WSI) dataset of breast cancer with annotated cell types hinders the ability of artificial intelligence to evaluate cell density in breast cancer WSI. We first employed Lasso-Cox regression to build a breast cancer prognosis assessment model based on cell density in a population study. Pathology experts manually annotated a dataset containing over 70,000 patches and used transfer learning based on ResNet152 to develop an artificial intelligence model for identifying different cell types in these patches. The results showed that significant prognostic differences were observed among breast cancer patients stratified by cell density score (*P* ═ 0.0018), with the cell density score identified as an independent prognostic factor for breast cancer patients (*P* < 0.05). In the validation cohort, the predictive performance for overall survival (OS) was satisfactory, with area under the curve (AUC) values of 0.893 (OS) at 1-year, 0.823 (OS) at 3-year, and 0.861 (OS) at 5-year intervals. We trained a robust model based on ResNet152, achieving over 99% classification accuracy for different cell types in patches. These achievements offer new public resources and tools for personalized treatment and prognosis assessment.

## Introduction

Breast cancer is the most common malignancy in women and a leading cause of cancer-related mortality [1]. In 2020, there were approximately 2.3 million new cases of breast cancer globally, accounting for 11.7% of all cancer incidence [2]. According to the International Agency for Research on Cancer, these numbers are expected to increase to over 3 million by 2040 [2, 3]. Actively exploring prognostic models for breast cancer is crucial, as these models not only predict disease progression and treatment outcomes for breast cancer patients, guiding physicians in determining appropriate treatment strategies but also provide corresponding risk assessment indicators to help patients better understand their disease status [4, 5]. However, current prognostic models based on tumor-related gene sets have limited clinical translation, as this would entail additional costs for patients for transcriptome sequencing and prolonged waiting periods for results.

Histopathological evaluation remains the gold standard for clinical diagnosis of breast cancer. Pathologic tissue sections are considered a multimodal source of information, containing tumor microenvironment (TME) data that can be used to reveal disease progression and predict patient prognosis [6]. TME plays a critical role in various stages of tumor progression [7, 8], and recent studies have indicated that the interactions between the TME and tumor cells determine tumor behavior, patient survival rates, and treatment responses in various cancers [9]. The breast TME is mainly composed of tumor cells, immune cells (myeloid cells, innate lymphoid cells, and lymphocytes), and stromal cells (fibroblasts and adipocytes), exhibiting complex dynamic interactions [10, 11]. Although the impact of tumor-infiltrating lymphocyte (TIL) density on patient outcomes has been studied [12, 13], a comprehensive assessment of the relationship between the densities of various cell types in the breast TME and prognosis is still lacking.

Although there are numerous prognostic factors in pathological tissue images, pathologists are unable to easily quantify cell density in histological images. This is because the manual analysis of cells in microscopic images is a time-consuming process, lacking objective standards and repeatability, and is thus prone to inter-observer variability [8, 14–16]. Deep learning represents the current state-of-the-art automated approach for handling histological images, and whole-slide imaging (WSI) has paved the way for integrating deep learning into the field of digital pathology [17]. However, the lack of high-quality annotated WSI datasets for breast cancer restricts the development of deep learning models [18–20]. The predictive performance of deep learning algorithms relies on the availability of sufficiently high-quality training and validation data [21, 22]. Currently, there is a lack of breast cancer WSI patch datasets annotated with cell types [21].

In this study, we investigated the independent impact of different types of cell density in the TME on the prognosis of breast cancer patients. A risk assessment model based on TME cell density (TMECD) was established and validated to predict the prognosis of breast cancer patients. We developed a patch-level multiclassification model for breast cancer cells based on deep convolutional neural networks (CNNs), aiming to assist clinical users in quickly and objectively obtaining cell density in breast cancer whole slide images (WSIs). As part of this work, we created a dataset containing over 70,000 patches of breast cancer WSI, which, to the best of our knowledge, is currently the only breast cancer patch-level WSI dataset annotated with cell types.

Our research findings indicate that the risk assessment model based on TMECD can effectively predict the prognosis of breast cancer patients. Additionally, the multiclassification model we developed demonstrates high accuracy and robustness in identifying different types of cells in breast cancer WSI. The availability of a large-scale, manually annotated patch-level WSI dataset also provides valuable resources for further investigations. These achievements offer new public resources and tools for personalized breast cancer treatment and prognosis assessment, with the potential to positively impact clinical practice.

## Materials and methods

### Data source

The anonymous pathological examination reports and pathological/frozen section information of breast cancer patients were obtained from the TCGA-BRCA cohort. The detailed information for each pathological/frozen section includes the percentage of lymphocyte infiltration (percent_LY), percentage of monocyte infiltration (percent_MO), percentage of necrosis (percent_NE), percentage of neutrophil infiltration (percent_NU), percentage of normal cells (percent_NO), percentage of stromal cells (percent_ST), percentage of tumor cells (percent_TU), and percentage of tumor nuclei (percent_TN). Furthermore, we manually calculated several additional features to comprehensively characterize the TME, including the percentage of immune cells (percent_IM ═ percent_LY + percent_MO + percent_NU), the ratio of tumor cells to stromal cells (TU_ST), the ratio of tumor cells to normal cells (TU_NO), the ratio of tumor cells to immune cells (TU_IM), the ratio of immune cells to stromal cells (IM_ST), and the ratio of immune cells to normal cells (IM_NO). After excluding patients with a survival time of less than 30 days and incomplete clinical information, a total of 362 patients were included in the subsequent study.

Using a similar approach, the anonymized pathological examination reports and pathological/frozen section information were retrieved from the TCGA-COAD, TCGA-LIHC, TCGA-LUAD, TCGA-LUSC, TCGA-PAAD, TCGA-STAD, and TCGA-THCA cohorts for additional analysis.

Forty WSIs from patients diagnosed with invasive breast cancer of no special type (IBC-NST) were used to develop deep CNNs to detect different types of breast cancer cells. These patients underwent surgical resection between August 2015 and August 2018. All enrolled patients provided written informed consent to use these samples for translational research, as approved by the Ethics Commission of the General Hospital of China PLA (approval number ky-2020-1-4). The study was compliant with the “Guidance of the Ministry of Science and Technology (MOST) for the Review and Approval of Human Genetic Resources,” China. All H&E-stained sections were scanned using a KFBIO Scans cope high-resolution scanner at 40× magnification (Konfoong Biotech, Ningbo, China) and digitized into KFB format.

### Image annotation and preprocessing

All regions of interest (ROIs) were manually marked by two experienced pathologists using QuPath (version: 0.4.0). Subsequently, the ROIs were cropped into patches of 256 × 256 pixels at a magnification of 40× to match the input scale of CNNs and avoid overfitting. Each patch was labeled as one of the following categories: adipose, immune cells, necrosis, normal breast cells, stromal cells, and tumor cells.

In constructing the training and validation datasets for the deep CNNs, oversampling was performed for immune cells, necrosis, and normal breast cells. This involved randomly applying one of the following operations to all patches and including them in the dataset along with the original image: rotation by 90∘ around the center, rotation by 180∘, and rotation by 270∘. Undersampling was applied to adipose, stromal cells, and tumor cells, involving the random selection of 3000 patches to be included in the dataset. The dataset used for training and testing the deep CNNs has been uploaded to Kaggle: https://www.kaggle.com/datasets/pupupu233/breast.

### Construction and validation of the prognostic model

The Lasso-based Cox regression was conducted using the R package glmnet (version 4.1–1). We employed L1 regularization and tenfold cross-validation and utilized stepwise regression to select the features used in the model. The formula for calculating the risk score is as follows: *RiskScore*═∑_i═1_^n^(β)× dens_i_, where dens*_i_* represents the density of feature *i*, and β*_i_* denotes the regression coefficient for the *i*th feature.

We categorized all patients into low-risk or high-risk groups based on the median of the risk scores derived from TMECD and performed survival analysis using the Kaplan–Meier method. The log-rank test was employed to compare the differences in survival status between the high-risk and low-risk groups. To assess the predictive capability of the risk scores based on TMECD, time-dependent receiver operating characteristic (ROC) curves were generated, and the area under the curve (AUC) for overall survival (OS) at one year, three years, and five years was calculated. The execution and visualization of Kaplan–Meier, log-rank, ROC curves, and calibration analysis were performed using the “survivalROC” (version 1.0.3), “rms” (version 6.2-0), “survival” (version 3.2-7), “survminer” (version 0.4.9), and “plotROC” (version 2.2.1) packages.

Furthermore, we conducted univariate and multivariate Cox regression analyses on the risk scores based on TMECD and other clinical features to confirm the independence of the risk scores as prognostic factors. Finally, we created a nomogram using the aforementioned features.

**Table 1:** Results of univariate cox regression analysis (*P* **<** 0.05)

**Variable**	**Beta**	**HR**	**HR lower**	**HR upper**	***P* value**
percent_TU	0.927255171	2.527561921	1.13006396	5.653281132	0.02396582
percent_LY	--0.226216788	0.797545185	0.652231472	0.975234022	0.027501115
TU_IM	0.185350084	1.203639742	1.001472277	1.446618804	0.048194948
percent_IM	--0.179390164	0.835779745	0.699476201	0.998644101	0.048279365

HR: Hazard ratio; percent_TU: Percentage of tumor cells; percent_LY: Percentage of lymphocyte infiltration; TU_IM: The ratio of tumor cells to immune cells; percent_IM: Percentage of immune cells.

### Training of deep CNNs

We employed transfer learning to train ResNet152 using the PyTorch framework (version 2.2.2). Pretrained parameters of ResNet152 on the ImageNet dataset were downloaded as initialization. The initial learning rate (LR) was set to 0.001, with stochastic gradient descent (SGD) utilized as the optimizer, momentum set to 0.9, and a batch size of 32. Furthermore, we reduced LR by a factor of 1/10 every 20 epochs. Normalization was performed using the mean (0.485, 0.456, 0.406) and standard deviation (0.229, 0.224, 0.225). The training process continued for 80 epochs, and the final model was saved. ACC and Loss were visualized using TensorBoard (version 2.16.2). The training was conducted utilizing a single GEFORCE RTX 4060Ti graphics card. The final model parameters obtained from the training process have been uploaded to Kaggle: https://www.kaggle.com/models/pupupu233/breast.

### Ethical statement

The study was conducted in accordance with the Declaration of Helsinki, and approved by the Ethics Commission of the General Hospital of China PLA (approval number ky-2020-1-4).

### Statistical analysis

Survival differences were assessed using the log-rank test, with *P* values < 0.05 considered statistically significant. The analysis was conducted using R (version 4.0.3). Accuracy (ACC), loss, and *F1* Score of the deep CNN were computed using Python (version 3.11.7).

## Results

### Construction and validation of breast cancer prognostic model based on cell density

As mentioned in the Methods section, we performed univariate Cox regression analysis on all features to investigate their independent contributions to prognosis. The results indicated that percent_TU, percent_LY, TU_IM, and percent_IM were significantly associated with patients’ survival outcomes (*P* < 0.05). Specifically, percent_TU and TU_IM were identified as adverse factors for patient outcomes, while percent_LY and percent_IM were recognized as protective factors. Notably, the maximum hazard ratio for percent_TU was 2.528, with a 95% CI of 1.130–5.653 (Table 1).

**Figure 1.**
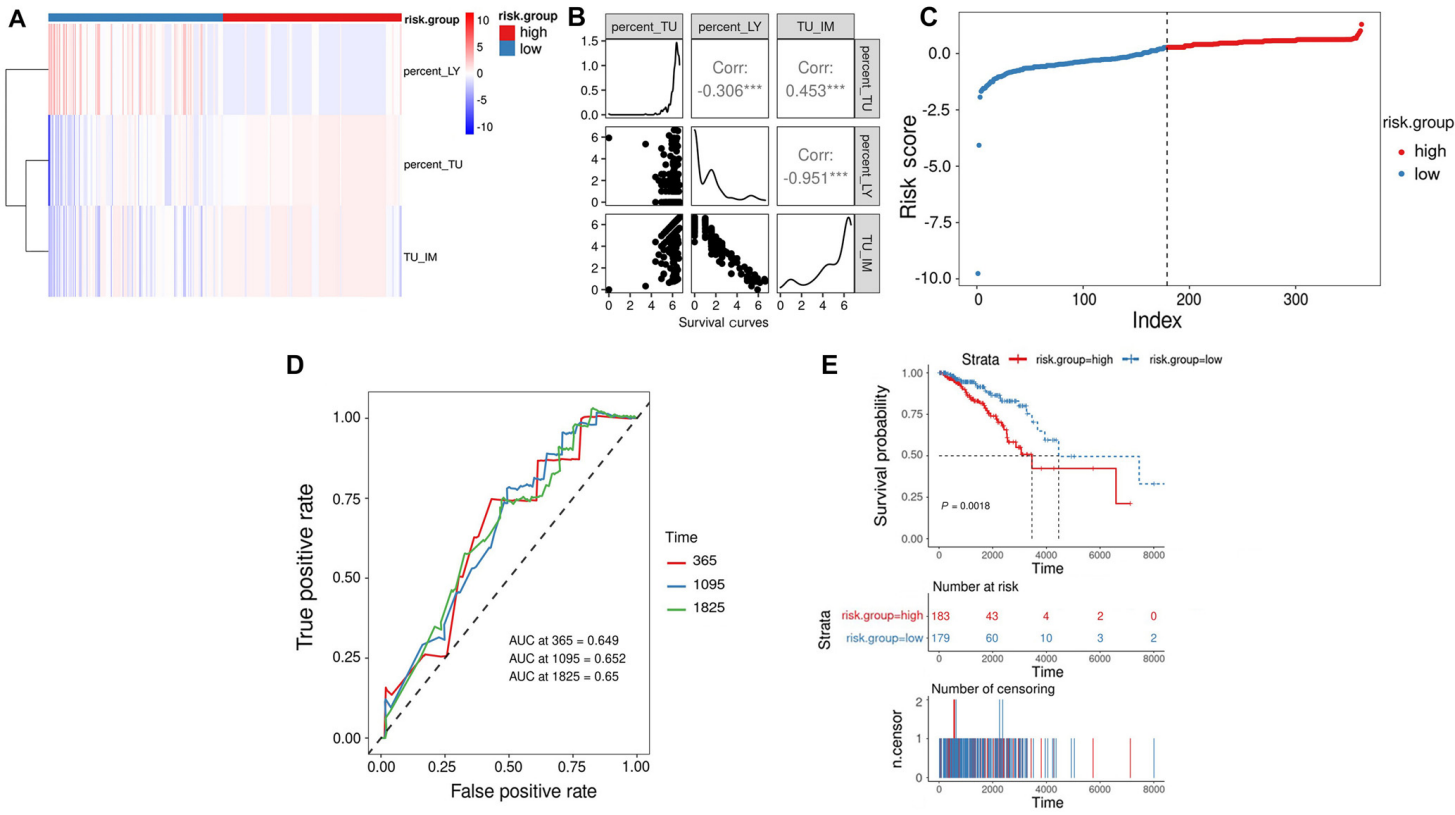
**A prognostic risk scoring model for breast cancer was developed based on the TMECD.** (A) A heatmap illustrating the distribution of the three cell density features among patients categorized into high- and low-risk groups; (B) The correlation of three cell density features within the TCGA-BRCA cohort; (C) Distribution of risk scores within the TCGA-BRCA cohort; (D) ROC curves and corresponding AUC values for 1-, 3-, and 5-year predictions based on TMECD risk features within the TCGA-BRCA cohort; (E) Kaplan–Meier survival analysis based on a validation cohort. AUC: Area under the curve; TMECD: Tumor microenvironment cell density; ROC: Receiver operating characteristic.

We conducted regression analysis using Lasso-Cox on four features, employing L1 regularization and ten-fold cross-validation, and utilized stepwise regression to minimize the number of features used by the model. Ultimately, we developed a risk assessment criterion based on TMECD to predict the OS of patients in the TCGA-BRCA cohort. The risk score formula is as follows: RiskScore ═ 1.5639 * percent_TU - 0.9927 * percent_LY - 0.8887 * TU_IM. We investigated the correlation among the three features in BRCA patients, revealing a significant positive correlation between percent_TU and TU_IM (correlation coefficient ═ 0.453, *P* < 0.01), a significant negative correlation between percent_TU and percent_LY (correlation coefficient ═ –0.306, *P* < 0.01), and a significant negative correlation between percent_LY and TU_IM (correlation coefficient ═ –0.951, *P* < 0.01) (Figure 1A and 1B). We computed risk scores for each BRCA patient and divided patients into high-risk and low-risk groups based on the median risk score (Figure 1C). Survival analysis using Kaplan–Meier curves revealed a significant association between the high-risk group and poorer OS, while the low-risk group was significantly associated with better OS (*P* ═ 0.0018, Figure 1D). To evaluate the predictive ability and accuracy of the risk model constructed based on TMECD, we plotted the time-dependent ROC curves with AUCs of 0.649, 0.652, and 0.65 for 1-year, 3-year, and 5-year predictions, respectively (Figure 1E).

### Development and validation of a model incorporating clinical features, along with corresponding nomogram

**Figure 2.**
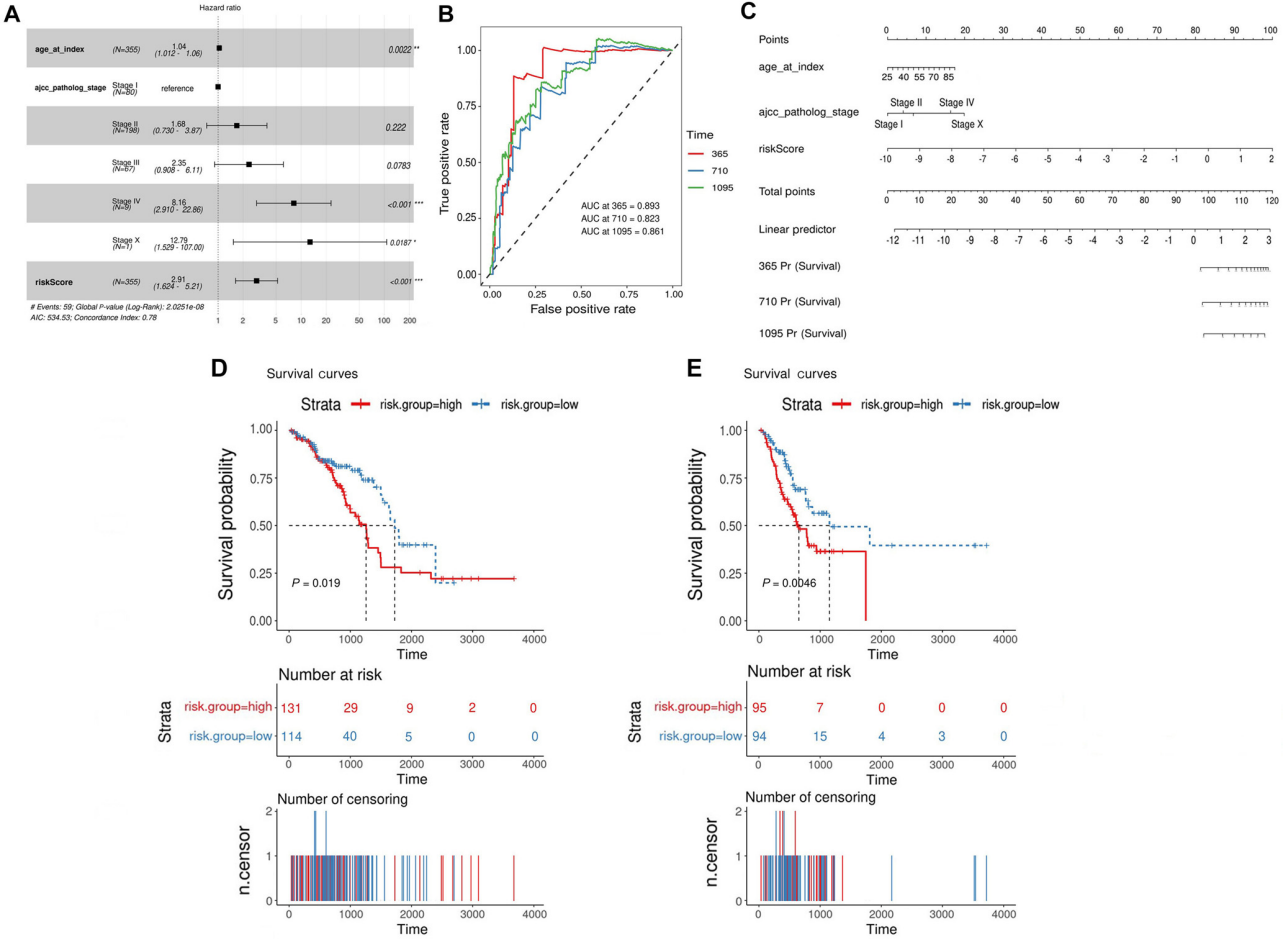
**Incorporating risk scoring based on clinical features and exploration in other cohorts.** (A) Risk ratio based on TMECD risk score and clinical features; (B) 1-, 3-, and 5-year predictive ROC curves and AUC for the prognostic model combining TMECD-based risk features and clinical features; (C) The nomogram constructed by integrating TMECD-based risk scores and clinical features; (D) Kaplan–Meier survival analysis of the prognostic model based on TMECD risk score in the TCGA-LUAD validation cohort; (E) Kaplan–Meier survival analysis of the prognostic model based on TMECD risk score in the TCGA-STAD validation cohort. AUC: Area under the curve; TMECD: Tumor microenvironment cell density; ROC: Receiver operating characteristic.

To investigate whether our risk score can serve as an independent prognostic factor for BRCA patients, we conducted a Cox regression analysis with patient risk scores based on TMECD, age, and AJCC pathological stage as covariates. The results indicated that the *P* value for the risk score feature based on TMECD was <0.001, confirming its utility in predicting the prognosis of BRCA patients (Figure 2A).

In order to enhance predictive performance, we integrated clinical indicators and constructed a risk feature combining TMECD risk scores with clinical parameters: RiskScoreII ═ 0.03442 * age + 0.51989 * AJCC pathological stage II + 0.85636 * AJCC pathological stage III + 2.09883 * AJCC pathological stage IV + 2.54872 * AJCC pathological stage X + 1.06779 * riskScore. Time-dependent ROC curves demonstrated that the AUCs for 1-year, 3-year, and 5-year predictions using the combined clinical and risk score features were 0.893, 0.823, and 0.861, respectively (Figure 2B), accompanied by a user-friendly nomogram for clinical application (Figure 2C).

As an additional investigation, we explored whether a TMECD-based risk score model could be established for other cancer types using the same approach. We attempted this analysis on seven common tumors, including TCGA-COAD, TCGA-LIHC, TCGA-LUAD, TCGA-LUSC, TCGA-PAAD, TCGA-STAD, and TCGA-THCA, but successfully developed prognostic models based on TMECD risk scores only for TCGA-LUAD and TCGA-STAD (Figure 2D and 2E).

### Construction of the breast cancer WSI patches dataset

**Figure 3.**
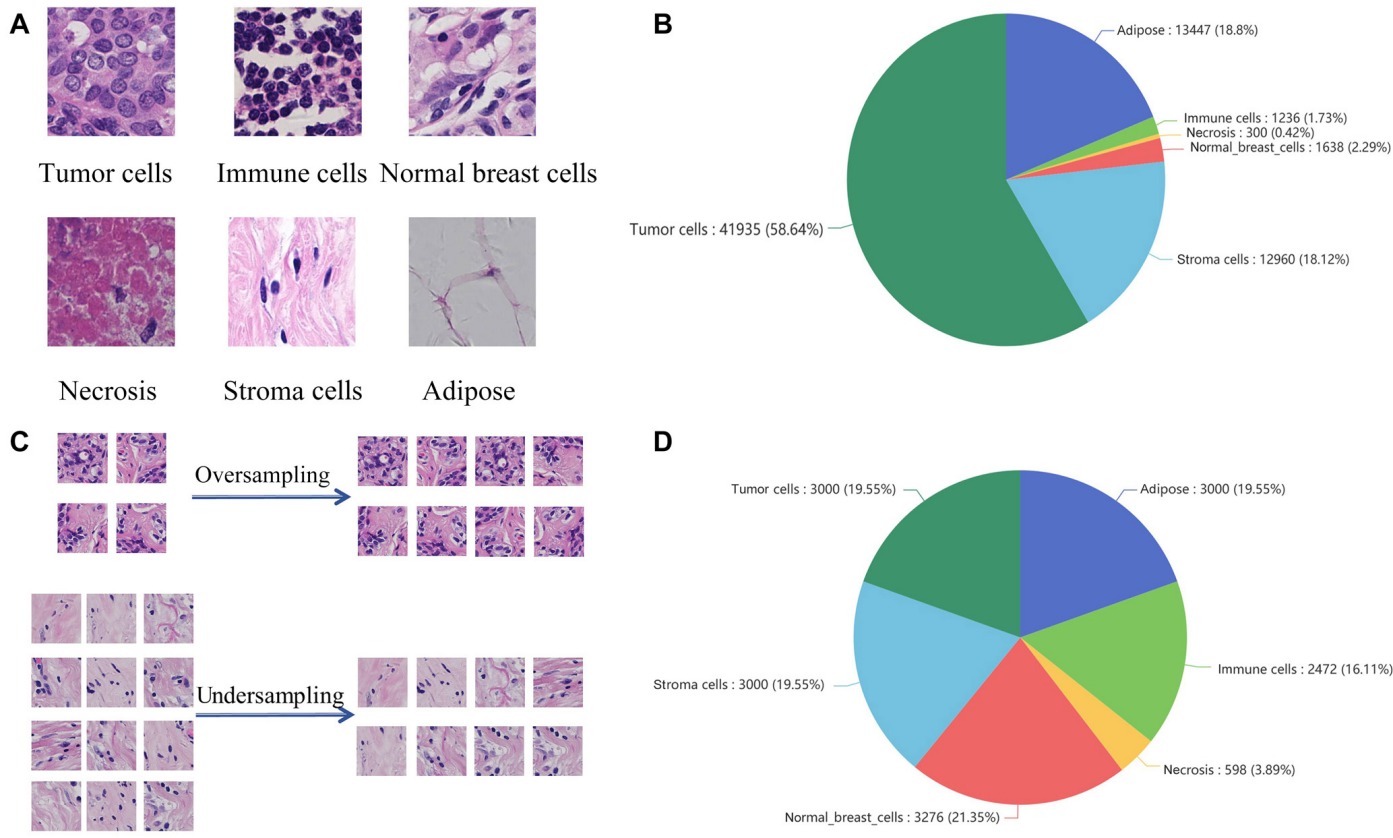
**Construction of a breast cancer WSI patch dataset.** (A) Examples of patches of different cell types; (B) Proportions of patches of different cell types before resampling; (C) The process of oversampling and undersampling; (D) Proportions of patches of different cell types after resampling. WSI: Whole slide image.

We collected 40 WSIs of IBC-NST to construct our dataset. All ROIs were delineated by two experienced pathologists using QuPath software. Subsequently, these ROIs were traversed and cropped into patches of 256 × 256 pixels at 40 × magnification. Each patch was annotated with the predominant histological type, determined based on the proportional area it occupied. We categorized patches into six types: adipose, immune cells, necrosis, normal breast cells, stromal cells, and tumor cells, resulting in a total of 71,516 patches with cell type annotations (Figure 3A).

Given the extreme imbalance in sample quantities across different cell types (Figure 3B), we performed data resampling to facilitate the training of deep learning models. Specifically, we oversampled immune cells, necrosis, and normal breast cells, while undersampling adipose, stromal cells, and tumor cells. This yielded a relatively balanced dataset for training and testing deep learning models (Figure 3C and 3D). The dataset used for training and testing the deep CNN has been uploaded to Kaggle: https://www.kaggle.com/datasets/pupupu233/breast.

### Training and validation of multiclass models based on transfer learning

**Figure 4.**
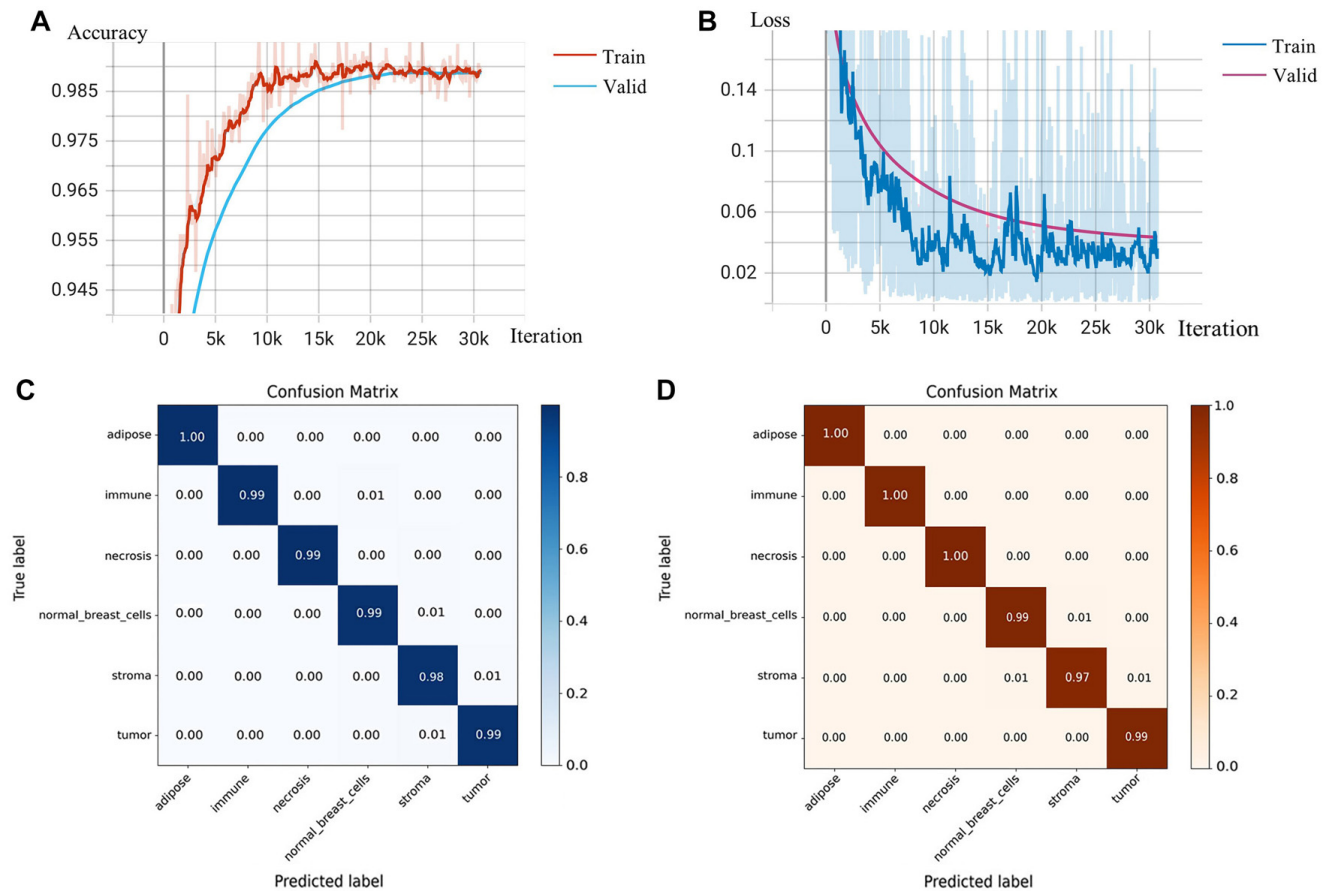
**Training and testing of deep CNNs.** (A) The variation of accuracy (ACC) of the deep CNN model on both the training and validation sets; (B) The variation of loss of the deep CNN model on both the training and validation sets; (C) The confusion matrix of the deep CNN model on the training set; (D) The confusion matrix of the deep CNN model on the validation set. CNNs: Convolutional neural networks.

To facilitate the clinical use of the TMECD-based risk scoring model for predicting patient prognosis, we developed a multiclassification model of breast cancer WSI cells using deep CNNs to objectively and rapidly quantify the density of different cells within breast cancer WSI. We fine-tuned the ResNet152 model, pretrained on ImageNet, on our local dataset to expedite convergence and improve classification performance. We set the BATCH_SIZE to 32 and the LR to 0.001, while reducing the LR by one-tenth every 20 epochs. After 80 epochs, the model’s accuracy (ACC) and loss on the test set stabilized, concluding the training process. At this point, the model achieved an ACC of 99.15% and reduced the loss to 0.0327, resulting in an F1 Score of 0.9929, indicating that the model’s precision meets the requirements (Figure 4A and 4B). The confusion matrix demonstrates the model’s high true-positive rate and extremely low false-negative and false-positive rates (Figure 4C and 4D). The final model parameters obtained from the training process have been uploaded to Kaggle: https://www.kaggle.com/models/pupupu233/breast.

## Discussion

Building a comprehensive dataset is crucial for the success of a deep learning project. To our knowledge, the dataset constructed in this study is currently the only available one with cell type annotations at the patch level for breast cancer WSI. The Camelyon 16 and 17 datasets are two well-known breast cancer WSI datasets provided by the 2016 Lymph Node Metastases Detection Challenge, consisting of approximately 2000 complete WSI images. However, only the entire WSI images are annotated for these datasets, indicating either normal tissue or malignant tumors. TCGA-BRCA could be the largest publicly available breast cancer WSI dataset, but it does not provide any usable region annotations. The NCT-CRC-HE-100K dataset is a widely cited colorectal cancer patch-level WSI dataset with cell type annotations, which has significantly advanced research in the field of colorectal cancer [23]. Based on this, we have constructed a large-scale patch-level WSI dataset for breast cancer, which includes annotations for various cell types (Table 2). This dataset aims to advance research in the digital pathology of breast cancer. The dataset used for training and testing the deep CNN in this study has been uploaded to Kaggle: https://www.kaggle.com/datasets/pupupu233/breast.

In this study, we investigated the independent impact of different types of cell densities in the TME on the prognosis of breast cancer patients and established and validated a risk assessment model based on TMECD to predict the prognosis of breast cancer patients. We found that percent_TU (percentage of tumor cells) and TU_IM (percentage of tumor cells/percentage of immune cells) were adverse factors for patient outcomes, while percent_LY (percentage of lymphocyte infiltration) and percent_IM (percentage of immune cells) played protective roles in patient prognosis (Table 1). The risk score based on TMECD was significantly correlated with the prognosis of breast cancer patients (Figure 2). To efficiently and objectively obtain the density of different types of cells from WSI images, we fine-tuned ResNet152 pretrained on the ImageNet dataset and trained a breast cancer WSI multicell classification model. On the validation set, the model achieved an ACC of 99.15%, a loss of 0.0327, and an *F1* Score of 0.9929. The model exhibited high true-positive rates and extremely low false-negative and false-positive rates (Figure 4). As part of this work, we created a breast cancer WSI patch dataset containing over 70,000 images annotated with cell types, which is currently the only breast cancer patch-level WSI dataset with cell type annotations. These achievements provide new avenues and tools for personalized breast cancer treatment and prognosis assessment, potentially playing a significant role in clinical applications.

**Table 2:** Comparison of the mentioned datasets

**Variable**	**Camelyon 16 and 17**	**TCGA-BRCA**	**NCT-CRC-HE-100K**	**Our datasets**
Source	The Netherlands	USA	Germany	China
Released	2016/2017	Not applicable	2019	2024
Public or not	Public	Public	Public	Public
Cell type annotations	No	No	Yes	Yes
Cancer	Breast cancer	Breast cancer	Colorectal cancer	Breast cancer

Breast cancer ranks among the most frequently diagnosed solid tumors in women, posing a serious threat to their physical and mental well-being [1, 24]. It is well recognized that the TME plays a crucial role in the growth, progression, and metastasis of tumors [25–29]. Studies have shown that TILs exert a favorable protective effect on the prognosis of breast cancer patients, which aligns with our findings [30–32]. The various cellular components within the TME of breast cancer exhibit intricate and dynamic interactions, with the stromal components likely playing a predominant role [33]. Among these, cancer-associated fibroblasts constitute an essential part of the stroma, sourced from a diverse array including normal fibroblasts, myofibroblasts, mesenchymal cells, stellate cells, fibrocytes, pericytes, smooth muscle cells, preadipocytes, and bone marrow-derived cells [34]. They manifest importance across various aspects of breast cancer, including growth, metastasis, response to treatment, and resistance to anticancer therapies [35]. In addition to stromal cell populations, diverse tumor-associated immune cells, such as tumor-associated neutrophils, tumor-associated lymphocytes, tumor-associated macrophages, dendritic cells, and mast cells augment the activation of stromal cells, thereby shaping the immunosuppressive TME [36]. Tumor-associated immune cells prevent the growth of immunoregulatory tumor cells by destroying them. However, they may also induce tumor resistance to therapy by influencing tumor immunogenicity and selecting for tumor clones capable of causing immune depletion [37]. Furthermore, immune cells within the TME exhibit dual functions in cancer development and metastasis. Th1-type helper T cells (Th1), cytotoxic T lymphocytes (CTLs), and natural killer cells (NK cells) are associated with an immunostimulatory microenvironment. In contrast, regulatory cells of the TME, including Th2-type helper T cells (Th2), tumor-associated macrophages, regulatory T cells (Tregs), and myeloid-derived suppressor cells, are associated with an immunosuppressive microenvironment and poor prognosis [38, 39]. These cells hinder tumor growth by eliminating immunoreactive tumor cells or altering tumor immunogenicity, thereby facilitating tumor escape [36].

ResNet-152 stands as one of the deepest architectures within the ResNet family, comprising a network structure with 152 layers, including a main backbone network and several auxiliary networks. The core idea of this architecture revolves around introducing residual connections, allowing information to propagate directly between network layers, thus alleviating the vanishing gradient problem. This enables the network to be trained deeper and more effectively. Its depth and performance make it excel on large-scale image datasets, enabling its application in various complex visual tasks [40]. It is worth mentioning that a multi-class classification model based on ResNet-152 achieved an ACC of 99.15% and an *F1* Score of 0.9929. The excellent performance of the multi-classifier is primarily attributed to advancements in deep learning algorithms. With the rapid development of deep neural network algorithms, AI models trained on large-scale data are poised to become valuable assistants for pathologists in pathology analysis. This advancement is expected to provide pathologists with more accurate and efficient diagnostic tools and potentially aid in uncovering and understanding hidden patterns and regularities within diseases [41, 42].

In the future, challenges in this field may focus on several key areas. Developing vertical large language models tailored to specific needs in digital pathology is beneficial; ChatGPT-4 has already been proven effective in maintaining high accuracy across various organizational pathology images [43]. Additionally, the development of interpretable artificial intelligence models is crucial as they would provide pathologists with sufficient evidence support [41]. On the data front, integrating multimodal imaging will offer more comprehensive disease information [44]. Simultaneously, expanding the coverage of datasets as much as possible will be a monumental task [45]. In terms of algorithms, ensemble learning could be a promising choice [46]. Finally, a comprehensive, all-in-one digital pathology analysis toolkit or cloud platform would significantly enhance convenience for clinical practitioners [47].

There are some limitations in our work. Firstly, the risk scores we constructed still require validation in another large-scale prospective pathology cohort. Additionally, deep CNNs need to be fine-tuned on multicenter datasets to ensure their stability when faced with data from different sources and in complex real-world scenarios.

## Conclusion

In conclusion, we have established and validated a breast cancer prognosis risk assessment model based on cell density, along with a patch-level artificial intelligence tool for identifying different cell types within breast cancer WSI. We manually annotated a dataset comprising over 70,000 patches, which, to our knowledge, is the only breast cancer patch-level dataset annotated with cell types. These achievements offer new insights and tools for personalized medicine and prognosis assessment.

## Data Availability

The dataset used for training and testing the deep CNNs has been uploaded to Kaggle: https://www.kaggle.com/datasets/pupupu233/breast. All patches are available from Kaggle: https://www.kaggle.com/datasets/pupupu233/all-patches.
